# Ethyl 23-benzyl-8,11,14-trioxa-23,28,29-triaza­penta­cyclo­[19.7.1.0^2,7^.0^15,20^.0^22,27^]nona­cosa-2,4,6,15(20),16,18,21,26-octa­ene-26-carboxyl­ate

**DOI:** 10.1107/S1600536813007241

**Published:** 2013-03-23

**Authors:** Truong Hong Hieu, Le Tuan Anh, Anatoly T. Soldatenkov, Vasily G. Vasil’ev, Victor N. Khrustalev

**Affiliations:** a Department of Chemistry, Vietnam National University, 144 Xuan Thuy, Cau Giay, Hanoi, Vietnam; b Organic Chemistry Department, Russian Peoples Friendship University, Miklukho-Maklaya St. 6, Moscow 117198, Russia; c X-Ray Structural Centre, A.N. Nesmeyanov Institute of Organoelement Compounds, Russian Academy of Sciences, 28 Vavilov St., B-334, Moscow 119991, Russian Federation

## Abstract

The title compound, C_33_H_35_N_3_O_5_, is the product of the multicomponent condensation of 1-benzyl-4-eth­oxy­carbonyl­piperidin-3-one with 1,5-bis­(2-formyl­phen­oxy)-3-oxapentane and ammonium acetate. The mol­ecule comprises a penta­cyclic system containing the aza-14-crown-4-ether macrocycle, tetra­hydro­pyrimidine, tetra­hydro­pyridine and two benzene rings. The aza-14-crown-4-ether ring adopts a bowl conformation with a dihedral angle of 62.37 (5)° between the benzene rings. The tetra­hydro­pyrimidine ring has an envelope conformation with the chiral C atom as the flap, whereas the tetra­hydro­pyridine ring adopts a distorted chair conformation. Two amino groups are involved in intra­molecular N—H⋯O hydrogen bonds. In the crystal, weak C—H⋯O hydrogen bonds link the mol­ecules into layers parallel to the *ab* plane.

## Related literature

For general background to the design, synthesis, chemical properties and applications of macrocyclic ligands for coordination chemistry, see: Hiraoka (1982 ▶); Pedersen (1988 ▶); Gokel & Murillo (1996 ▶); Bradshaw & Izatt (1997 ▶). For the crystal structures of related compounds, see: Levov *et al.* (2006 ▶, 2008 ▶); Komarova *et al.* (2008 ▶); Anh *et al.* (2008 ▶, 2012*a* ▶,*b* ▶,*c* ▶); Hieu *et al.* (2009 ▶, 2011 ▶, 2012*a* ▶,*b* ▶); Khieu *et al.* (2011 ▶); Sokol *et al.* (2011 ▶).
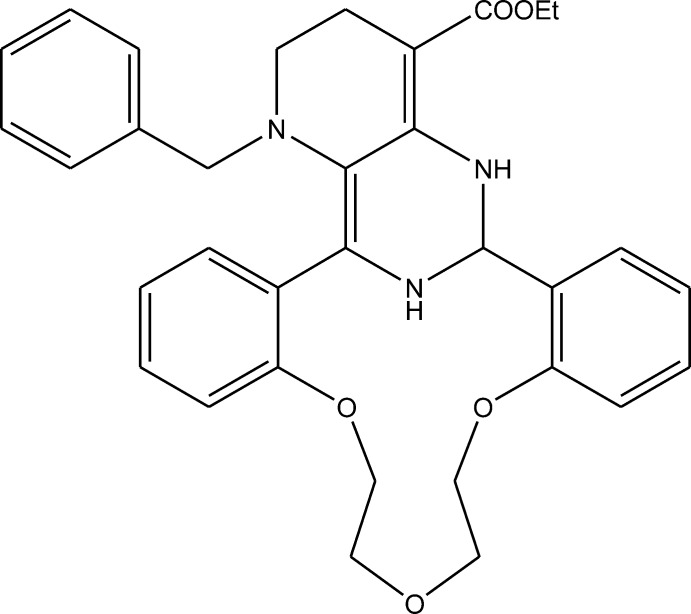


## Experimental

### 

#### Crystal data

C_33_H_35_N_3_O_5_
*M* *_r_* = 553.64
Monoclinic, 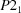

*a* = 10.5304 (5) Å
*b* = 12.6363 (5) Å
*c* = 10.7246 (5) Å
β = 92.865 (1)°
*V* = 1425.29 (11) Å^3^
*Z* = 2
Mo *K*α radiation
μ = 0.09 mm^−1^
*T* = 100 K
0.30 × 0.24 × 0.21 mm

#### Data collection

Bruker APEXII CCD diffractometer
Absorption correction: multi-scan (*SADABS*; Sheldrick, 2003 ▶) *T*_min_ = 0.974, *T*_max_ = 0.982
18837 measured reflections
8289 independent reflections
6878 reflections with *I* > 2σ(*I*)
*R*_int_ = 0.027

#### Refinement

*R*[*F* ^2^ > 2σ(*F* ^2^)] = 0.040
*wR*(*F* ^2^) = 0.094
*S* = 1.00
8289 reflections
377 parameters
1 restraint
H atoms treated by a mixture of independent and constrained refinement
Δρ_max_ = 0.23 e Å^−3^
Δρ_min_ = −0.18 e Å^−3^

### 

Data collection: *APEX2* (Bruker, 2005 ▶); cell refinement: *SAINT* (Bruker, 2001 ▶); data reduction: *SAINT*; program(s) used to solve structure: *SHELXTL* (Sheldrick, 2008 ▶); program(s) used to refine structure: *SHELXTL*; molecular graphics: *SHELXTL*; software used to prepare material for publication: *SHELXTL*.

## Supplementary Material

Crystal structure: contains datablock(s) global, I. DOI: 10.1107/S1600536813007241/cv5393sup1.cif

Structure factors: contains datablock(s) I. DOI: 10.1107/S1600536813007241/cv5393Isup2.hkl

Supplementary material file. DOI: 10.1107/S1600536813007241/cv5393Isup3.cml

Additional supplementary materials:  crystallographic information; 3D view; checkCIF report

## Figures and Tables

**Table 1:** Hydrogen-bond geometry (Å, °)

*D*—H⋯*A*	*D*—H	H⋯*A*	*D*⋯*A*	*D*—H⋯*A*
N24—H24⋯O1′	0.882 (18)	2.015 (18)	2.6928 (16)	132.8 (15)
N25—H25⋯O14	0.882 (18)	2.441 (17)	2.9744 (17)	119.3 (13)
C6—H6⋯O1′^i^	0.95	2.42	3.3516 (19)	168
C18—H18⋯O1′^ii^	0.95	2.42	3.3613 (19)	174

Symmetry codes: (i) 
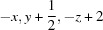
; (ii) 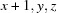.

**Fig. 1.**
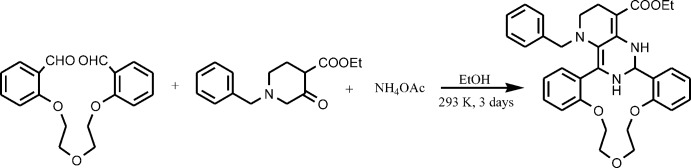
Multicomponent condensation of the 1-benzyl-4-ethoxycarbonylpiperidin-3-one with 1,5-bis(2-formylphenoxy)-3-oxapentane and ammonium acetate.

**Fig. 2.**
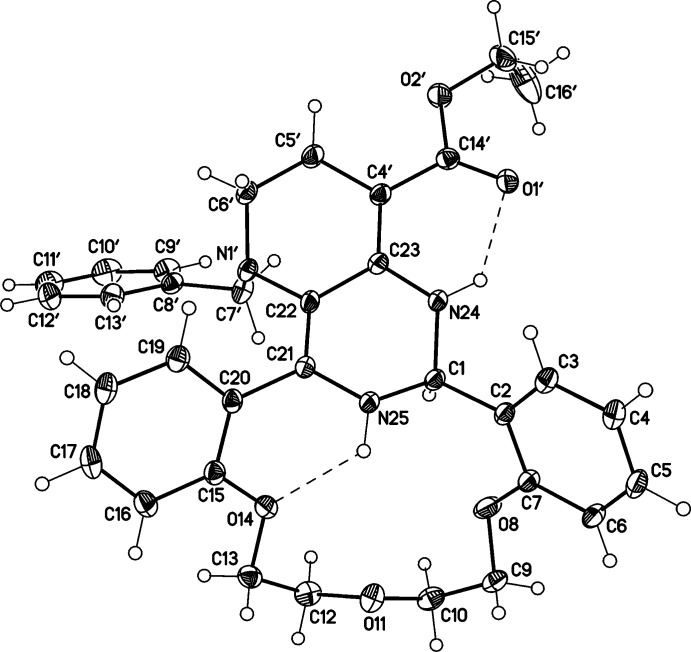
Molecular structure of I. Displacement ellipsoids are shown at the 50% probability level. H atoms are presented as small spheres of arbitrary radius. The intramolecular N—H···O hydrogen bonds are drawn by dashed lines.

**Fig. 3.**
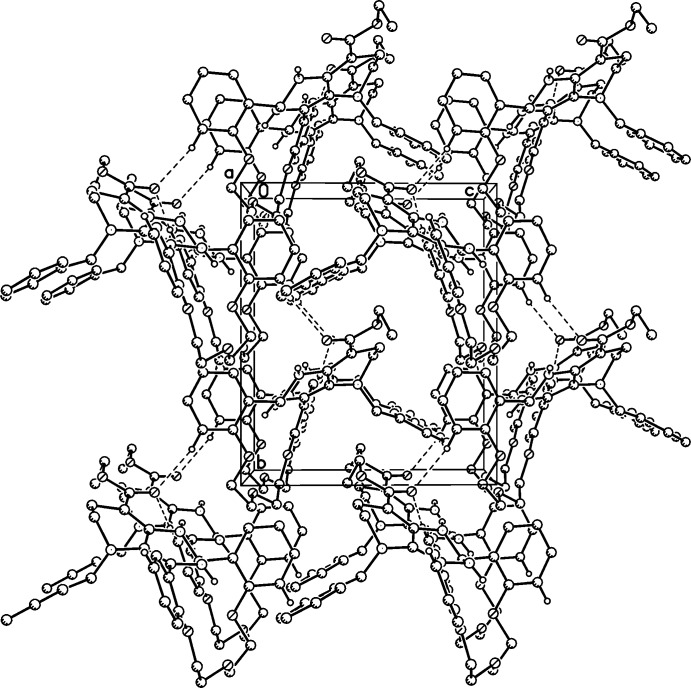
A portion of the crystal structure showing the weak intermolecular C—H···O hydrogen bonds, which are depicted by dashed lines.

## supplementary crystallographic information

### Comment

Design, preparation and applications of macroheterocyclic ligands for coordination, supramolecular and medicinal chemistry draw constant attention of investigators during the last several decades (Hiraoka, 1982; Pedersen,1988; Gokel & Murillo, 1996; Bradshaw & Izatt, 1997). Recently we have developed the effective method of synthesis of azacrown ethers including piperidine (Levov *et al.*, 2006); Anh *et al.*, 2008, 2012*a*,*b*), cycloalkanopiperidine (Levov *et al.* 2008); bispidine (Komarova *et al.*; Sokol *et al.*; Hieu *et al.* 2012*a*; Anh *et al.* 2012*c*); perhydropyrimidine (Hieu *et al.*, 2011) and perhydrotriazine (Hieu *et al.*, 2009, 2012*b*; Khieu *et al.*, 2011) subunits.

In attempts to apply the chemistry for obtaining azacrown ether containing ethoxy-substituted bispidino subunit with two nitrogen atoms in the unsymmetrical positions, we studied the multicomponent condensation of the 1-benzyl-4-ethoxycarbonylpiperidin-3-one (ketone component) with 1,5-bis(2-formylphenoxy)-3-oxapentane (podand) and ammonium acetate. The reaction has proceeded smoothly under mild conditions to give the title compound as an unexpected product (Fig. 1). The first step of this cascade process appears to be the intermolecular condensation of one aldehyde group of the podand with the activated methylene group of the ketone component. Then the addition of one molecule of ammonia to the keto-group yields its hydroxyl-amino form. Further the second aldehyde group is condensed with the amino group to form the intermediate azacrown ether containing 1,4-azadiene fragment fused to piperidine moiety. The final step is the double Mannich cycloaddition of another molecule of ammonia to the azadiene moiety followed by dehydration to form the product. The structure of the new azacrown system, C_33_H_35_N_3_O_5_ (I), was unambiguously established by X-ray diffraction study.

The molecule of I comprises a pentacyclic system containing the aza-14-crown-4-ether macrocycle, tetrahydropyrimidine, tetrahydropyridine and two benzene rings (Fig. 2). The aza-14-crown-4-ether ring adopts a bowl conformation. The configuration of the C7—O8—C9—C10 —O11—C12—C13—O14—C15 polyether chain is t–g^(-)^–t–t–g^(+)^–t (t = *trans*, 180°; g = *gauche*, ±60°). The dihedral angle between the planes of the benzene rings fused to the aza-14-crown-4-ether moiety is 62.37 (5)°. The central tetrahydropyrimidine ring has an envelope conformation (the C1 carbon atom is out of the plane passed through the other atoms of the ring (r.m.s. deviation = 0.023 Å) by 0.661 (2) Å), which is stabilized by the intramolecular N25—H25···O14 hydrogen bond (Table 1). The terminal tetrahydropyridine ring adopts a distorted *chair* conformation (the N1' nitrogen and C6' carbon atoms are out of the plane passed through the other atoms of the ring (r.m.s. deviation = 0.012 Å) by -0.245 (3) and 0.431 (3) Å, respectively). The three N24, N25 and N1' nitrogen atoms have the trigonal-pyramidal geometries. The carboxylate substituent (except for the terminal C16' carbon atom) is practically coplanar to the basal C22—C23—C4'—C5' plane of the tetrahydropyridine ring (the O2'—C14'—C4'—C5' dihedral angle is -5.5 (2)°). This disposition is apparently determined by the intramolecular N24—H24···O1' hydrogen bond (Table 1).

The molecule of I possesses an asymmetric center at the C1 carbon atom and crystallizes in the chiral space group *P*2_1_. However, its absolute configuration cannot be objectively determined because the absence of the heavy (*Z* > 14) atoms within the molecule.

In the crystal, the molecules of I are bound by the weak intermolecular C—H···O hydrogen bonding interactions (Table 1) into layers parallel to *ab>* plane (Figure 3).

### Experimental

Ammonium acetate (5.0 g, 65 mmol) was added to a solution of 1,5-bis(2-formylphenoxy)-3-oxapentane (1.57 g, 5.0 mmol) and 1-benzyl-4-ethoxycarbonylpiperidin-3-one (1.48 g, 5.0 mmol) in ethanol (30 ml) – acetic acid (2 ml). The reaction mixture was stirred at 293 K for 3 days. At the end of the reaction, the formed precipitate was filtered off, washed with ethanol and chromatographically purified on the column filled with silica gel. A re-crystallization from hexane:ethylacetate (3:1) mixture gave 0.83 g of light-yellow crystals of I.Yield is 30.0%. *M*.p. = 373–376 K. IR (KBr), ν/cm^-1^: 1599, 1644, 3297, 3374, 3453. ^1^H NMR (CDCl_3_, 400 MHz, 300 K): δ = 1.29 (t, 3H, *J* = 7.2 and 6.8, CH_2_CH_3_), 2.26 and 2.78 (both m, 1H and 3H, correspondingly, NCH_2_CH_2_), 3.50 and 3.85 (both d, 1H each, *J* = 13.2 each, NCH_2_*Ar*), 3.73–4.15 (m, 9H, OCH_2_CH_2_OCH_2_CH_2_O and CH_2_CH_3_), 4.83 (s, 1H, N—H25), 6.05 (s, 1H, H1), 6.73 (dd, 2H, *J* = 7.7 and 1.6, H6 and H16), 6.8 (broad t, 2H, *J* = 8.9, H4 and H18), 6.97–7.09 (m, 5H, H_arom_), 7.28–7.32 (m, 2H, H_arom_), 7.47 (dd, 2H, *J* = 7.6 and 1.6, H3), 7.87 (dd, 2H, *J* = 7.6 and 1.2, H19), 8.61 (s, 1H, N—H24). Anal. Calcd for C_33_H_35_N_3_O_5_: C, 71.59; H, 6.37; N, 7.59. Found: C, 71.53; H, 6.22; N, 7.37.

### Refinement

The absolute structure of I cannot be objectively determined by the refinement of Flack parameter because the absence of the heavy (*Z* > 14) atoms within the molecule.

The hydrogen atoms of the amino groups were localized in the difference-Fourier map and refined isotropically with fixed isotropic displacement parameters [*U*_iso_(H) = 1.2*U*_eq_(N)]. The other hydrogen atoms were placed in calculated positions with C—H = 0.95–1.00 Å and refined in the riding model with fixed isotropic displacement parameters [*U*_iso_(H) = 1.5*U*_eq_(C) for the methyl group and 1.2*U*_eq_(C) for the other groups].

### Crystal data

**Table:**

C_33_H_35_N_3_O_5_	*F*(000) = 588
*M**_r_* = 553.64	*D*_x_ = 1.290 Mg m^−^^3^
Monoclinic, *P*2_1_	Mo *K*α radiation, λ = 0.71073 Å
Hall symbol: P 2yb	Cell parameters from 5715 reflections
*a* = 10.5304 (5) Å	θ = 2.5–31.8°
*b* = 12.6363 (5) Å	µ = 0.09 mm^−^^1^
*c* = 10.7246 (5) Å	*T* = 100 K
β = 92.865 (1)°	Prism, yellow
*V* = 1425.29 (11) Å^3^	0.30 × 0.24 × 0.21 mm
*Z* = 2	

### Data collection

**Table:**

Bruker APEXII CCD diffractometer	8289 independent reflections
Radiation source: fine-focus sealed tube	6878 reflections with *I* > 2σ(*I*)
Graphite monochromator	*R*_int_ = 0.027
φ and ω scans	θ_max_ = 30.0°, θ_min_ = 1.9°
Absorption correction: multi-scan (*SADABS*; Sheldrick, 2003)	*h* = −14→14
*T*_min_ = 0.974, *T*_max_ = 0.982	*k* = −17→17
18837 measured reflections	*l* = −15→15

### Refinement

**Table:**

Refinement on *F*^2^	Primary atom site location: structure-invariant direct methods
Least-squares matrix: full	Secondary atom site location: difference Fourier map
*R*[*F*^2^ > 2σ(*F*^2^)] = 0.040	Hydrogen site location: difference Fourier map
*wR*(*F*^2^) = 0.094	H atoms treated by a mixture of independent and constrained refinement
*S* = 1.00	*w* = 1/[σ^2^(*F*_o_^2^) + (0.0431*P*)^2^ + 0.123*P*] where *P* = (*F*_o_^2^ + 2*F*_c_^2^)/3
8289 reflections	(Δ/σ)_max_ < 0.001
377 parameters	Δρ_max_ = 0.23 e Å^−^^3^
1 restraint	Δρ_min_ = −0.18 e Å^−^^3^

### Special details

**Table:**

Geometry. All e.s.d.'s (except the e.s.d. in the dihedral angle between two l.s. planes) are estimated using the full covariance matrix. The cell e.s.d.'s are taken into account individually in the estimation of e.s.d.'s in distances, angles and torsion angles; correlations between e.s.d.'s in cell parameters are only used when they are defined by crystal symmetry. An approximate (isotropic) treatment of cell e.s.d.'s is used for estimating e.s.d.'s involving l.s. planes.
Refinement. Refinement of *F*^2^ against ALL reflections. The weighted *R*-factor *wR* and goodness of fit *S* are based on *F*^2^, conventional *R*-factors *R* are based on *F*, with *F* set to zero for negative *F*^2^. The threshold expression of *F*^2^ > σ(*F*^2^) is used only for calculating *R*-factors(gt) *etc*. and is not relevant to the choice of reflections for refinement. *R*-factors based on *F*^2^ are statistically about twice as large as those based on *F*, and *R*- factors based on ALL data will be even larger.

### Fractional atomic coordinates and isotropic or equivalent isotropic displacement parameters (Å^2^)

**Table:**

	*x*	*y*	*z*	*U*_iso_*/*U*_eq_	
C1	0.22606 (12)	0.22945 (12)	0.85547 (12)	0.0266 (3)	
H1	0.1992	0.2950	0.8090	0.032*	
C2	0.16248 (12)	0.22544 (12)	0.97887 (12)	0.0271 (3)	
C3	0.13663 (14)	0.13054 (13)	1.03713 (13)	0.0310 (3)	
H3	0.1570	0.0660	0.9974	0.037*	
C4	0.08137 (14)	0.12737 (14)	1.15267 (14)	0.0347 (3)	
H4	0.0644	0.0615	1.1912	0.042*	
C5	0.05172 (14)	0.22147 (15)	1.21021 (14)	0.0376 (3)	
H5	0.0148	0.2200	1.2892	0.045*	
C6	0.07508 (15)	0.31771 (14)	1.15432 (14)	0.0365 (3)	
H6	0.0533	0.3819	1.1942	0.044*	
C7	0.13097 (13)	0.31993 (13)	1.03861 (13)	0.0312 (3)	
O8	0.15789 (13)	0.41119 (10)	0.97691 (11)	0.0441 (3)	
C9	0.16598 (16)	0.50775 (12)	1.04698 (14)	0.0360 (3)	
H9A	0.2106	0.4957	1.1292	0.043*	
H9B	0.0799	0.5356	1.0607	0.043*	
C10	0.23872 (16)	0.58408 (13)	0.97119 (14)	0.0367 (3)	
H10A	0.2017	0.5874	0.8846	0.044*	
H10B	0.2362	0.6558	1.0081	0.044*	
O11	0.36498 (10)	0.54650 (9)	0.97231 (10)	0.0380 (3)	
C12	0.43821 (16)	0.59053 (13)	0.87902 (14)	0.0372 (3)	
H12A	0.4594	0.6651	0.8995	0.045*	
H12B	0.3899	0.5889	0.7975	0.045*	
C13	0.55719 (15)	0.52654 (12)	0.87280 (14)	0.0338 (3)	
H13A	0.6134	0.5571	0.8108	0.041*	
H13B	0.6039	0.5252	0.9552	0.041*	
O14	0.51863 (10)	0.42183 (9)	0.83650 (11)	0.0372 (2)	
C15	0.61093 (14)	0.35024 (12)	0.81031 (13)	0.0298 (3)	
C16	0.74063 (14)	0.37340 (13)	0.81731 (15)	0.0361 (3)	
H16	0.7694	0.4404	0.8472	0.043*	
C17	0.82748 (14)	0.29860 (14)	0.78062 (15)	0.0388 (4)	
H17	0.9156	0.3150	0.7847	0.047*	
C18	0.78697 (14)	0.20051 (13)	0.73815 (15)	0.0367 (4)	
H18	0.8465	0.1499	0.7114	0.044*	
C19	0.65764 (14)	0.17656 (13)	0.73505 (14)	0.0325 (3)	
H19	0.6300	0.1085	0.7075	0.039*	
C20	0.56790 (13)	0.25002 (12)	0.77133 (12)	0.0273 (3)	
C21	0.43077 (12)	0.22008 (12)	0.76601 (12)	0.0269 (3)	
C22	0.37341 (13)	0.17558 (12)	0.66288 (13)	0.0272 (3)	
C23	0.24979 (13)	0.12495 (11)	0.66886 (12)	0.0255 (3)	
N24	0.19281 (12)	0.13681 (10)	0.78031 (11)	0.0289 (3)	
H24	0.1137 (17)	0.1140 (14)	0.7817 (16)	0.035*	
N25	0.36503 (11)	0.22816 (11)	0.87544 (11)	0.0278 (2)	
H25	0.3951 (16)	0.2792 (14)	0.9247 (16)	0.033*	
N1'	0.43614 (11)	0.17458 (10)	0.54726 (10)	0.0272 (2)	
C4'	0.20023 (13)	0.06475 (12)	0.57082 (12)	0.0280 (3)	
C5'	0.27720 (14)	0.04519 (14)	0.45751 (13)	0.0353 (3)	
H5A	0.2425	0.0885	0.3869	0.042*	
H5B	0.2699	−0.0302	0.4331	0.042*	
C6'	0.41709 (14)	0.07292 (12)	0.48401 (13)	0.0306 (3)	
H6A	0.4575	0.0165	0.5364	0.037*	
H6B	0.4602	0.0747	0.4041	0.037*	
C7'	0.39864 (15)	0.26627 (13)	0.46972 (15)	0.0357 (3)	
H7A	0.3881	0.3282	0.5248	0.043*	
H7B	0.3151	0.2514	0.4269	0.043*	
C8'	0.49217 (14)	0.29427 (11)	0.37288 (13)	0.0301 (3)	
C9'	0.44865 (17)	0.34402 (13)	0.26331 (15)	0.0399 (4)	
H9	0.3599	0.3525	0.2462	0.048*	
C10'	0.5333 (2)	0.38131 (15)	0.17872 (17)	0.0510 (5)	
H10	0.5024	0.4164	0.1049	0.061*	
C11'	0.6611 (2)	0.36766 (15)	0.20134 (18)	0.0542 (5)	
H11	0.7189	0.3930	0.1431	0.065*	
C12'	0.70637 (18)	0.31703 (15)	0.30879 (18)	0.0474 (4)	
H12	0.7952	0.3070	0.3239	0.057*	
C13'	0.62174 (15)	0.28063 (13)	0.39512 (15)	0.0366 (3)	
H13	0.6531	0.2464	0.4693	0.044*	
C14'	0.07571 (14)	0.01805 (12)	0.57658 (13)	0.0298 (3)	
O1'	0.00688 (10)	0.02090 (9)	0.66562 (9)	0.0342 (2)	
O2'	0.03869 (11)	−0.03237 (11)	0.46902 (11)	0.0452 (3)	
C15'	−0.08813 (17)	−0.07689 (17)	0.46202 (18)	0.0507 (5)	
H15A	−0.1111	−0.1000	0.5462	0.061*	
H15B	−0.0901	−0.1397	0.4068	0.061*	
C16'	−0.1818 (2)	0.0022 (2)	0.4129 (3)	0.0858 (9)	
H16A	−0.2654	−0.0313	0.4006	0.129*	
H16B	−0.1548	0.0298	0.3330	0.129*	
H16C	−0.1871	0.0605	0.4727	0.129*	

### Atomic displacement parameters (Å^2^)

**Table:**

	*U*^11^	*U*^22^	*U*^33^	*U*^12^	*U*^13^	*U*^23^
C1	0.0254 (6)	0.0334 (7)	0.0211 (6)	0.0014 (6)	0.0027 (5)	−0.0038 (5)
C2	0.0213 (6)	0.0390 (7)	0.0210 (6)	0.0017 (6)	0.0012 (5)	−0.0044 (6)
C3	0.0274 (7)	0.0388 (8)	0.0271 (7)	0.0060 (6)	0.0032 (5)	−0.0015 (6)
C4	0.0281 (7)	0.0466 (9)	0.0299 (7)	0.0044 (6)	0.0053 (6)	0.0039 (6)
C5	0.0307 (7)	0.0566 (10)	0.0262 (7)	0.0022 (7)	0.0087 (5)	−0.0027 (7)
C6	0.0354 (8)	0.0456 (9)	0.0295 (7)	0.0015 (7)	0.0089 (6)	−0.0103 (7)
C7	0.0298 (7)	0.0385 (8)	0.0257 (7)	−0.0007 (6)	0.0045 (5)	−0.0063 (6)
O8	0.0656 (8)	0.0368 (6)	0.0311 (6)	−0.0050 (6)	0.0139 (5)	−0.0102 (5)
C9	0.0405 (8)	0.0359 (8)	0.0317 (7)	0.0062 (6)	0.0044 (6)	−0.0091 (6)
C10	0.0431 (9)	0.0337 (8)	0.0329 (8)	0.0110 (7)	−0.0015 (6)	−0.0025 (6)
O11	0.0395 (6)	0.0419 (6)	0.0330 (5)	0.0087 (5)	0.0049 (5)	0.0092 (5)
C12	0.0484 (9)	0.0305 (7)	0.0327 (8)	0.0005 (7)	0.0014 (7)	0.0052 (6)
C13	0.0386 (8)	0.0323 (8)	0.0306 (7)	−0.0076 (6)	0.0026 (6)	0.0011 (6)
O14	0.0305 (5)	0.0331 (6)	0.0484 (7)	−0.0034 (4)	0.0060 (5)	−0.0088 (5)
C15	0.0286 (7)	0.0345 (7)	0.0267 (7)	−0.0018 (6)	0.0049 (5)	0.0024 (5)
C16	0.0315 (8)	0.0398 (9)	0.0370 (8)	−0.0073 (6)	0.0027 (6)	0.0032 (7)
C17	0.0251 (7)	0.0517 (10)	0.0399 (8)	−0.0032 (7)	0.0043 (6)	0.0146 (7)
C18	0.0283 (7)	0.0469 (10)	0.0353 (8)	0.0096 (6)	0.0059 (6)	0.0089 (7)
C19	0.0322 (7)	0.0368 (8)	0.0288 (7)	0.0029 (6)	0.0035 (6)	0.0033 (6)
C20	0.0252 (6)	0.0358 (8)	0.0211 (6)	−0.0013 (5)	0.0034 (5)	0.0027 (5)
C21	0.0244 (6)	0.0309 (7)	0.0257 (6)	−0.0001 (6)	0.0046 (5)	−0.0027 (6)
C22	0.0266 (7)	0.0336 (7)	0.0220 (6)	−0.0012 (6)	0.0070 (5)	−0.0004 (5)
C23	0.0260 (6)	0.0287 (7)	0.0220 (6)	0.0024 (5)	0.0026 (5)	−0.0005 (5)
N24	0.0261 (6)	0.0375 (7)	0.0237 (5)	−0.0049 (5)	0.0061 (5)	−0.0066 (5)
N25	0.0242 (5)	0.0367 (6)	0.0229 (5)	−0.0029 (5)	0.0039 (4)	−0.0067 (5)
N1'	0.0292 (6)	0.0331 (6)	0.0197 (5)	0.0018 (5)	0.0057 (4)	0.0014 (4)
C4'	0.0285 (7)	0.0341 (8)	0.0216 (6)	0.0000 (6)	0.0020 (5)	−0.0031 (5)
C5'	0.0343 (8)	0.0487 (9)	0.0230 (6)	−0.0027 (7)	0.0043 (6)	−0.0086 (6)
C6'	0.0305 (7)	0.0380 (8)	0.0236 (6)	0.0027 (6)	0.0058 (5)	−0.0037 (6)
C7'	0.0349 (8)	0.0405 (8)	0.0323 (7)	0.0109 (6)	0.0092 (6)	0.0078 (6)
C8'	0.0372 (8)	0.0272 (7)	0.0265 (7)	0.0017 (6)	0.0064 (6)	0.0013 (5)
C9'	0.0499 (9)	0.0385 (9)	0.0311 (7)	0.0039 (7)	0.0001 (7)	0.0061 (6)
C10'	0.0774 (14)	0.0410 (10)	0.0352 (9)	−0.0028 (9)	0.0092 (9)	0.0128 (7)
C11'	0.0757 (14)	0.0426 (10)	0.0466 (10)	−0.0157 (9)	0.0262 (10)	0.0039 (8)
C12'	0.0409 (9)	0.0468 (10)	0.0555 (10)	−0.0095 (8)	0.0138 (8)	−0.0014 (9)
C13'	0.0377 (8)	0.0378 (8)	0.0346 (8)	0.0006 (6)	0.0047 (6)	0.0034 (6)
C14'	0.0307 (7)	0.0312 (7)	0.0273 (7)	−0.0001 (6)	−0.0015 (5)	−0.0019 (6)
O1'	0.0327 (5)	0.0388 (6)	0.0314 (5)	−0.0058 (4)	0.0052 (4)	−0.0018 (4)
O2'	0.0381 (6)	0.0640 (8)	0.0335 (6)	−0.0157 (6)	0.0017 (5)	−0.0158 (5)
C15'	0.0457 (10)	0.0629 (12)	0.0430 (10)	−0.0233 (9)	−0.0026 (8)	−0.0109 (9)
C16'	0.0484 (12)	0.113 (2)	0.0931 (19)	−0.0321 (13)	−0.0237 (12)	0.0484 (17)

### Geometric parameters (Å, º)

**Table:**

C1—N24	1.4542 (18)	C21—C22	1.3556 (19)
C1—N25	1.4685 (17)	C21—N25	1.3957 (16)
C1—C2	1.5135 (17)	C22—N1'	1.4338 (17)
C1—H1	1.0000	C22—C23	1.4548 (19)
C2—C3	1.386 (2)	C23—N24	1.3721 (17)
C2—C7	1.403 (2)	C23—C4'	1.3790 (19)
C3—C4	1.396 (2)	N24—H24	0.882 (18)
C3—H3	0.9500	N25—H25	0.883 (18)
C4—C5	1.383 (2)	N1'—C6'	1.4620 (19)
C4—H4	0.9500	N1'—C7'	1.4687 (19)
C5—C6	1.383 (3)	C4'—C14'	1.442 (2)
C5—H5	0.9500	C4'—C5'	1.5144 (19)
C6—C7	1.400 (2)	C5'—C6'	1.527 (2)
C6—H6	0.9500	C5'—H5A	0.9900
C7—O8	1.366 (2)	C5'—H5B	0.9900
O8—C9	1.4334 (18)	C6'—H6A	0.9900
C9—C10	1.497 (2)	C6'—H6B	0.9900
C9—H9A	0.9900	C7'—C8'	1.509 (2)
C9—H9B	0.9900	C7'—H7A	0.9900
C10—O11	1.4114 (19)	C7'—H7B	0.9900
C10—H10A	0.9900	C8'—C13'	1.384 (2)
C10—H10B	0.9900	C8'—C9'	1.390 (2)
O11—C12	1.4079 (18)	C9'—C10'	1.386 (2)
C12—C13	1.495 (2)	C9'—H9	0.9500
C12—H12A	0.9900	C10'—C11'	1.367 (3)
C12—H12B	0.9900	C10'—H10	0.9500
C13—O14	1.4322 (18)	C11'—C12'	1.382 (3)
C13—H13A	0.9900	C11'—H11	0.9500
C13—H13B	0.9900	C12'—C13'	1.395 (2)
O14—C15	1.3672 (18)	C12'—H12	0.9500
C15—C16	1.395 (2)	C13'—H13	0.9500
C15—C20	1.402 (2)	C14'—O1'	1.2279 (17)
C16—C17	1.386 (2)	C14'—O2'	1.3578 (17)
C16—H16	0.9500	O2'—C15'	1.448 (2)
C17—C18	1.381 (2)	C15'—C16'	1.482 (3)
C17—H17	0.9500	C15'—H15A	0.9900
C18—C19	1.394 (2)	C15'—H15B	0.9900
C18—H18	0.9500	C16'—H16A	0.9800
C19—C20	1.394 (2)	C16'—H16B	0.9800
C19—H19	0.9500	C16'—H16C	0.9800
C20—C21	1.4912 (18)		
			
N24—C1—N25	106.41 (11)	N25—C21—C20	117.99 (11)
N24—C1—C2	110.67 (12)	C21—C22—N1'	120.23 (12)
N25—C1—C2	110.68 (11)	C21—C22—C23	120.64 (12)
N24—C1—H1	109.7	N1'—C22—C23	119.09 (12)
N25—C1—H1	109.7	N24—C23—C4'	123.99 (12)
C2—C1—H1	109.7	N24—C23—C22	114.94 (12)
C3—C2—C7	118.32 (12)	C4'—C23—C22	120.95 (12)
C3—C2—C1	121.92 (13)	C23—N24—C1	117.85 (12)
C7—C2—C1	119.74 (13)	C23—N24—H24	115.7 (11)
C2—C3—C4	121.69 (14)	C1—N24—H24	117.0 (11)
C2—C3—H3	119.2	C21—N25—C1	114.25 (11)
C4—C3—H3	119.2	C21—N25—H25	112.2 (11)
C5—C4—C3	119.02 (15)	C1—N25—H25	113.9 (11)
C5—C4—H4	120.5	C22—N1'—C6'	110.52 (11)
C3—C4—H4	120.5	C22—N1'—C7'	111.12 (11)
C4—C5—C6	120.91 (13)	C6'—N1'—C7'	113.80 (11)
C4—C5—H5	119.5	C23—C4'—C14'	120.22 (12)
C6—C5—H5	119.5	C23—C4'—C5'	120.30 (12)
C5—C6—C7	119.57 (14)	C14'—C4'—C5'	119.46 (12)
C5—C6—H6	120.2	C4'—C5'—C6'	111.30 (11)
C7—C6—H6	120.2	C4'—C5'—H5A	109.4
O8—C7—C6	123.56 (14)	C6'—C5'—H5A	109.4
O8—C7—C2	115.95 (12)	C4'—C5'—H5B	109.4
C6—C7—C2	120.49 (14)	C6'—C5'—H5B	109.4
C7—O8—C9	118.22 (11)	H5A—C5'—H5B	108.0
O8—C9—C10	106.41 (12)	N1'—C6'—C5'	113.37 (12)
O8—C9—H9A	110.4	N1'—C6'—H6A	108.9
C10—C9—H9A	110.4	C5'—C6'—H6A	108.9
O8—C9—H9B	110.4	N1'—C6'—H6B	108.9
C10—C9—H9B	110.4	C5'—C6'—H6B	108.9
H9A—C9—H9B	108.6	H6A—C6'—H6B	107.7
O11—C10—C9	106.62 (12)	N1'—C7'—C8'	114.11 (12)
O11—C10—H10A	110.4	N1'—C7'—H7A	108.7
C9—C10—H10A	110.4	C8'—C7'—H7A	108.7
O11—C10—H10B	110.4	N1'—C7'—H7B	108.7
C9—C10—H10B	110.4	C8'—C7'—H7B	108.7
H10A—C10—H10B	108.6	H7A—C7'—H7B	107.6
C12—O11—C10	114.21 (12)	C13'—C8'—C9'	118.85 (14)
O11—C12—C13	107.93 (12)	C13'—C8'—C7'	121.60 (13)
O11—C12—H12A	110.1	C9'—C8'—C7'	119.27 (14)
C13—C12—H12A	110.1	C10'—C9'—C8'	120.77 (16)
O11—C12—H12B	110.1	C10'—C9'—H9	119.6
C13—C12—H12B	110.1	C8'—C9'—H9	119.6
H12A—C12—H12B	108.4	C11'—C10'—C9'	120.05 (17)
O14—C13—C12	106.55 (12)	C11'—C10'—H10	120.0
O14—C13—H13A	110.4	C9'—C10'—H10	120.0
C12—C13—H13A	110.4	C10'—C11'—C12'	120.14 (16)
O14—C13—H13B	110.4	C10'—C11'—H11	119.9
C12—C13—H13B	110.4	C12'—C11'—H11	119.9
H13A—C13—H13B	108.6	C11'—C12'—C13'	120.06 (17)
C15—O14—C13	118.19 (12)	C11'—C12'—H12	120.0
O14—C15—C16	123.66 (14)	C13'—C12'—H12	120.0
O14—C15—C20	115.89 (12)	C8'—C13'—C12'	120.10 (15)
C16—C15—C20	120.43 (14)	C8'—C13'—H13	119.9
C17—C16—C15	119.99 (15)	C12'—C13'—H13	119.9
C17—C16—H16	120.0	O1'—C14'—O2'	121.26 (13)
C15—C16—H16	120.0	O1'—C14'—C4'	126.49 (13)
C18—C17—C16	120.57 (14)	O2'—C14'—C4'	112.25 (12)
C18—C17—H17	119.7	C14'—O2'—C15'	116.83 (12)
C16—C17—H17	119.7	O2'—C15'—C16'	110.61 (17)
C17—C18—C19	119.21 (14)	O2'—C15'—H15A	109.5
C17—C18—H18	120.4	C16'—C15'—H15A	109.5
C19—C18—H18	120.4	O2'—C15'—H15B	109.5
C20—C19—C18	121.60 (15)	C16'—C15'—H15B	109.5
C20—C19—H19	119.2	H15A—C15'—H15B	108.1
C18—C19—H19	119.2	C15'—C16'—H16A	109.5
C19—C20—C15	118.12 (13)	C15'—C16'—H16B	109.5
C19—C20—C21	119.28 (13)	H16A—C16'—H16B	109.5
C15—C20—C21	122.59 (12)	C15'—C16'—H16C	109.5
C22—C21—N25	119.80 (12)	H16A—C16'—H16C	109.5
C22—C21—C20	121.84 (12)	H16B—C16'—H16C	109.5
			
N24—C1—C2—C3	30.99 (17)	C21—C22—C23—N24	6.9 (2)
N25—C1—C2—C3	−86.73 (16)	N1'—C22—C23—N24	−175.27 (12)
N24—C1—C2—C7	−150.56 (13)	C21—C22—C23—C4'	−169.29 (14)
N25—C1—C2—C7	91.72 (15)	N1'—C22—C23—C4'	8.5 (2)
C7—C2—C3—C4	−0.4 (2)	C4'—C23—N24—C1	−157.44 (13)
C1—C2—C3—C4	178.09 (13)	C22—C23—N24—C1	26.47 (18)
C2—C3—C4—C5	0.0 (2)	N25—C1—N24—C23	−55.37 (16)
C3—C4—C5—C6	0.6 (2)	C2—C1—N24—C23	−175.67 (12)
C4—C5—C6—C7	−0.8 (2)	C22—C21—N25—C1	−24.73 (19)
C5—C6—C7—O8	−179.74 (15)	C20—C21—N25—C1	162.10 (13)
C5—C6—C7—C2	0.4 (2)	N24—C1—N25—C21	53.10 (16)
C3—C2—C7—O8	−179.70 (13)	C2—C1—N25—C21	173.39 (12)
C1—C2—C7—O8	1.80 (18)	C21—C22—N1'—C6'	139.13 (14)
C3—C2—C7—C6	0.1 (2)	C23—C22—N1'—C6'	−38.69 (17)
C1—C2—C7—C6	−178.37 (13)	C21—C22—N1'—C7'	−93.54 (16)
C6—C7—O8—C9	19.4 (2)	C23—C22—N1'—C7'	88.65 (16)
C2—C7—O8—C9	−160.75 (13)	N24—C23—C4'—C14'	6.2 (2)
C7—O8—C9—C10	160.67 (13)	C22—C23—C4'—C14'	−177.91 (13)
O8—C9—C10—O11	−68.86 (15)	N24—C23—C4'—C5'	−172.46 (14)
C9—C10—O11—C12	163.21 (13)	C22—C23—C4'—C5'	3.4 (2)
C10—O11—C12—C13	−167.25 (13)	C23—C4'—C5'—C6'	15.1 (2)
O11—C12—C13—O14	62.76 (15)	C14'—C4'—C5'—C6'	−163.57 (13)
C12—C13—O14—C15	172.76 (12)	C22—N1'—C6'—C5'	57.81 (15)
C13—O14—C15—C16	0.2 (2)	C7'—N1'—C6'—C5'	−68.03 (15)
C13—O14—C15—C20	−177.99 (12)	C4'—C5'—C6'—N1'	−46.07 (17)
O14—C15—C16—C17	−175.38 (15)	C22—N1'—C7'—C8'	158.26 (13)
C20—C15—C16—C17	2.7 (2)	C6'—N1'—C7'—C8'	−76.22 (16)
C15—C16—C17—C18	−0.7 (2)	N1'—C7'—C8'—C13'	−34.0 (2)
C16—C17—C18—C19	−1.4 (2)	N1'—C7'—C8'—C9'	152.10 (14)
C17—C18—C19—C20	1.4 (2)	C13'—C8'—C9'—C10'	−1.3 (2)
C18—C19—C20—C15	0.7 (2)	C7'—C8'—C9'—C10'	172.83 (16)
C18—C19—C20—C21	179.96 (13)	C8'—C9'—C10'—C11'	1.2 (3)
O14—C15—C20—C19	175.56 (13)	C9'—C10'—C11'—C12'	−0.3 (3)
C16—C15—C20—C19	−2.7 (2)	C10'—C11'—C12'—C13'	−0.6 (3)
O14—C15—C20—C21	−3.7 (2)	C9'—C8'—C13'—C12'	0.4 (2)
C16—C15—C20—C21	178.04 (13)	C7'—C8'—C13'—C12'	−173.59 (16)
C19—C20—C21—C22	−49.5 (2)	C11'—C12'—C13'—C8'	0.6 (3)
C15—C20—C21—C22	129.79 (16)	C23—C4'—C14'—O1'	−4.2 (2)
C19—C20—C21—N25	123.54 (15)	C5'—C4'—C14'—O1'	174.51 (14)
C15—C20—C21—N25	−57.19 (19)	C23—C4'—C14'—O2'	175.85 (13)
N25—C21—C22—N1'	174.95 (13)	C5'—C4'—C14'—O2'	−5.5 (2)
C20—C21—C22—N1'	−12.2 (2)	O1'—C14'—O2'—C15'	3.5 (2)
N25—C21—C22—C23	−7.3 (2)	C4'—C14'—O2'—C15'	−176.51 (15)
C20—C21—C22—C23	165.62 (13)	C14'—O2'—C15'—C16'	88.9 (2)

### Hydrogen-bond geometry (Å, º)

**Table:**

*D*—H···*A*	*D*—H	H···*A*	*D*···*A*	*D*—H···*A*
N24—H24···O1′	0.882 (18)	2.015 (18)	2.6928 (16)	132.8 (15)
N25—H25···O14	0.882 (18)	2.441 (17)	2.9744 (17)	119.3 (13)
C6—H6···O1′^i^	0.95	2.42	3.3516 (19)	168
C18—H18···O1′^ii^	0.95	2.42	3.3613 (19)	174

Symmetry codes: (i) −*x*, *y*+1/2, −*z*+2; (ii) *x*+1, *y*, *z*.
